# Evidence for decline in the incidence of cystic fibrosis: a 35-year observational study in Brittany, France

**DOI:** 10.1186/1750-1172-7-14

**Published:** 2012-03-01

**Authors:** Virginie Scotet, Ingrid Duguépéroux, Philippe Saliou, Gilles Rault, Michel Roussey, Marie-Pierre Audrézet, Claude Férec

**Affiliations:** 1 Inserm, U1078, Brest, F-29200, France; 2 Univ Brest, Brest, F-29200, France; 3 Etablissement Français du Sang-Bretagne, Brest, F-29200, France; 4 CHRU Brest, Hôp. Morvan, Laboratoire de Génétique Moléculaire, Brest, F-29200, France; 5 Centre de ressources et de compétences de la mucoviscidose, Roscoff, F-29680, France; 6 Centre de ressources et de compétences de la mucoviscidose, Rennes, F-35000, France; 7 Association Française pour le Dépistage et la Prévention des Handicaps de l'Enfant (AFDPHE), Paris, F-75000, France

**Keywords:** Cystic fibrosis, Incidence, Time trends, Newborn screening, Pregnancy ultrasound examination

## Abstract

**Background:**

Cystic fibrosis (CF) is an autosomal recessive disorder whose incidence has long been estimated as 1/2500 live births in Caucasians. Expanding implementation of newborn screening (NBS) programs now allows a better monitoring of the disease incidence, what is essential to make reliable predictions for disease management. This study assessed time trends in the birth incidence of CF over a long period (35 years: 1975-2009) in an area where CF is frequent (Brittany, France) and where NBS has been implemented for more than 20 years.

**Methods:**

This study enrolled CF patients born in Brittany between January 1^st ^1975 and December 31^st ^2009 (n = 483). Time trends in incidence were examined using Poisson regression and mainly expressed using the average percent change (APC).

**Results:**

The average number of patients born each year declined from 18.6 in the late 1970's (period 1975-79) to 11.6 nowadays (period 2005-09). The corresponding incidence rates dropped from 1/1983 to 1/3268, which represented a decline close to 40% between these two periods (APC = -39.3%, 95% CI = -55.8% to -16.7%, p = 0.0020). A clear breakpoint in incidence rate was observed at the end of the 1980's (p < 0.0001). However, the incidence rate has remained quite stable since that time (annual APC = -1.0%, 95% CI = -3.0% to 1.1%, p = 0.3516).

**Conclusions:**

This study provides an accurate picture of the evolution of the incidence of a genetic disease over a long period and highlights how it is influenced by the health policies implemented. We observed a 40% drop in incidence in our area which seems consecutive to the availability of prenatal diagnosis.

## Background

Cystic fibrosis (CF; OMIM#219700) is an autosomal recessive disorder due to mutations in the *CFTR *(*Cystic Fibrosis Transmembrane conductance Regulator*) gene, which was cloned in 1989 [1]. Newborn screening (NBS) for CF, which has progressively been implemented worldwide over the past years, now allows better monitoring of the birth incidence of CF, which appears lower than in the past. Long estimated as 4.0/10,000 (i.e. 1/2500) live births in Caucasians [2], the average birth incidence rate is now 2.9/10,000 (i.e. 1/3500) in Europe [3,4].

Time trends in the birth incidence of CF have been investigated in several studies, but the magnitude of the reported changes varies according to the areas and to the length of the period under study [5-8]. The trends observed result from a complex mixture of phenomena and have been ascribed to various health policies (as prenatal diagnosis, family testing [9], population-based carrier screening [10-12]) and/or to demographic factors (as reduction of family size, increase of population mixing with consecutive decline of endogamy and inbreeding [8]).

Time trends in the birth incidence therefore depend on the health policies implemented in the studied population, but also on the characteristics of the population (i.e. carrier rate, cultural attitudes toward prenatal diagnosis and pregnancy termination, birth rate, etc). Monitoring of CF incidence is therefore essential to be able to make reliable predictions for disease management in the future.

In this study, we examined time trends in the incidence of CF over a long period (the longest ever studied to date: 35 years, 1975-2009) in an area where CF is frequent (Brittany, western France), where NBS has been implemented for a long time (since 1989) but where carrier screening (both preconceptional screening and prenatal screening) is not underway.

## Methods

### Study population

This study enrolled CF patients born in Brittany (western France) between January 1^st ^1975 and December 31^st ^2009. Confined at the western end of Europe, Brittany is a region of three million inhabitants where the incidence of CF is among the highest in the world [13] and where an NBS program has been under way since 1989 [14]. This particular situation led us to set up a registry of molecular epidemiology of CF in this area, in order to analyze temporal trends in incidence and survival [13].

Brittany has a relatively homogeneous population, which mainly results from historical waves of migrations of Celtic people. The geographical structure of this area (peninsula) coupled with the use, for many centuries, of a Celtic language (the Breton) have contributed to the isolation of its population. This resulted in low populations' mixings and high levels of endogamy and inbreeding.

### Data sources

The patients included in the present study were extracted from this registry, which also gathers newborn screening and prenatal diagnosis data. As previously described [13], patients born before the set up of NBS were retrieved through active enquiries and a combination of data sources (with capture-recapture studies). Patients born since the implementation of NBS were easily collected by consulting data from the French organization in charge of this program (A.F.D.P.H.E.) as well as data from the genetics laboratories of our area.

In order to ensure data homogeneity, the same inclusion criteria were used throughout. Patients with an equivocal diagnosis that could have been detected through NBS were excluded from the analysis (i.e. newborns screened with ambiguous sweat test results and/or *CFTR *mutations not clearly associated with CF, as for example those carrying the R117H: n = 19) [15-17]. All patients born during the study period were enrolled, including siblings as well as false-negative cases missed by our NBS program.

### Statistical analysis

Statistical analysis was performed using SAS software (version 9.2-SAS Institute, Cary, NC, USA). The significance level was set at *p *≤ 5% for all analyses.

#### Birth incidence rate calculation

The birth incidence rate of CF (with its 95% confidence interval-95% CI) was determined for the whole study period, by 5-year periods and by year. The incidence rate of a given year was calculated by dividing the number of CF children born over that year by:

1) *Prior to the implementation of NBS: *the number of live births that occurred in Brittany over that year (data provided by the French Institute of Statistics and Economic Studies).

2) *After the implementation of NBS: *the number of screening tests performed in Brittany over that year (data provided by the organization in charge of the NBS program). It is important to note that the refusal rate for this test is extremely low in our area (< 0.01%).

#### Time trends analysis

Time trends in the incidence rates were examined using Poisson regression, which enables to model count or rate data. This approach, which derives from generalized linear models [18], is classically used to analyze time trends in incidence of cancers or of chronic diseases.

The analyses were conducted by considering the whole study period, but also 5-year periods as well as other periods of interest (defined by the time of implementation of prenatal diagnosis or newborn screening). For all analyses, the oldest period was taken as reference.

The changes observed in the incidence rates were expressed by two parameters:

1) the odds-ratio (OR) with its 95% CI. The OR corresponds to the exponential of the β coefficient that estimates the effect of time. It indicates how much the incidence rate observed over a period has decreased in comparison with the reference period.

2) the average percent change (APC) with its 95% CI. This parameter is derived from the formula ([exp(β)-1]*100) and represents the percentage of variation observed in the incidence rates between the periods of interest ([Incidence _Period_-Incidence _Reference period_]/Incidence _Reference period_). When time is coded as the year of birth, the APC represents the annual average percent change in the incidence rate.

All the models were fitted using the "Proc genmod" procedure in the SAS software. A constant rate of change was assumed over the period concerned (log-linearity assumption) and the presence of under/over dispersion was checked by introducing the term "scale = pearson" in the models.

#### Adjusted incidence rate calculation

Prenatal diagnosis of CF was developed in the early 1980's [19,20]. Beyond the parents of CF child(ren), prenatal diagnosis is offered to the 1-in-4 risk couples identified through family testing, but also, since the early 1990's, to the 1-in-4 risk couples identified following the *in utero *detection of a sonographic sign suggestive of CF (echogenic bowel). If the fetus is found to be CF-affected, genetic counseling is offered to the couple and the possibility of therapeutic termination is made available.

As we have knowledge of the number of pregnancy terminations performed yearly after a positive prenatal diagnosis (made by molecular biology) in our area, we were also able to calculate an adjusted incidence rate (by adding these data to the calculations) and therefore to assess the impact of prenatal diagnosis on the incidence rate. We also examined in detail the contexts in which the 1-in-4 risk was discovered (i.e. the contexts in which the PD was done), and assessed the impact of each of these contexts on the incidence rate.

This research protocol was approved by the *Comité Consultatif sur le Traitement de l'Information en Matière de Recherche dans le domaine de la Santé *and by the *Commission Nationale d'Informatique et des Libertés*.

## Results

### Birth incidence rate and its time trends

A total of 483 CF children born in Brittany between January 1^st ^1975 and December 31^st ^2009 were recorded. Half of them (n = 243, 50.3%) were born since the implementation of the NBS program in 1989. The *CFTR *genotype could be fully determined in 96.5% of the patients (n = 466) and 56.0% of them (n = 261) were homozygous for the main F508del (p.Phe508del) mutation. These data led to a global CF birth incidence rate of 3.8/10,000 (95% CI = 3.4/10,000 to 4.2/10,000) in Brittany over the study period (i.e. 1/2637).

**Figure 1:**
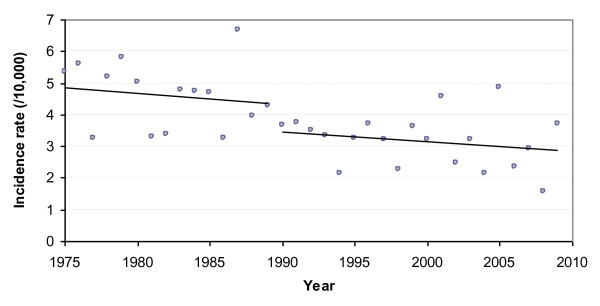
**Evolution of the annual birth incidence rate of cystic fibrosis in Brittany over the period 1975-2009**. As statistical analysis revealed that the most significant change in incidence rate was observed when the year 1990 was considered as the cut-off time, we plotted the trendlines independently for the periods 1975-1989 and 1990-2009.

Figure 1 shows the evolution of the annual birth incidence rate of CF over the 35-year period. Despite year-to-year variations, Poisson regression analysis revealed a significant decrease in the incidence rate over the whole study period (p < 0.0001). The annual APC was -1.8% (95% CI = -2.6% to -1.0%), meaning that the incidence rate decreased on average by 1.8% each year.

The analysis performed by 5-year periods (Tables 1 and 2) indicated that the incidence rate dropped from 5.0/10,000 (i.e. 1/1983) in the late 1970's to 3.1/10,000 (i.e. 1/3268). The average number of CF children born each year over these two periods decreased from 18.6 to 11.6.

**Table 1:** Birth incidence rates of cystic fibrosis in Brittany by 5-year period (period 1975-2009).

Period	No. patients	No. births	Incidence (95% CI) (1/xxxx)	Incidence (95% CI) (/10,000)
**1975-1979**	93	184407	**1/1983 **(1/2413; 1/1629)	**5.0 **(4.1; 6.1)
**1980-1984**	80	189746	**1/2372 **(1/3880; 1/1450)	**4.2 **(2.6; 6.9)
**1985-1989**	82	179446	**1/2188 **(1/3573; 1/1340)	**4.6 **(2.8; 7.4)
**1990-1994**	56	169926	**1/3034 **(1/5123; 1/1797)	**3.3 **(2.0; 5.5)
**1995-1999**	56	174412	**1/3115 **(1/5258; 1/1845)	**3.2 **(1.9; 5.4)
**2000-2004**	58	186054	**1/3208 **(1/5396; 1/1907)	**3.1 **(1.9; 5.2)
**2005-2009**	58	189563	**1/3268 **(1/5498; 1/1943)	**3.1 **(1.8; 5.1)

**Table 2:** Results of the time trend analysis: odds-ratio (OR) and average percent change (APC) associated with each 5-year period (period 1975-2009).

Period	Incidence (1/xxxx)	Incidence (/10,000)	OR (95% CI) e^β^	APC (95% CI) (e^β^-1)*100	*p*
**1975-1979**	**1/1983**	**5.0**	**ref**.	**ref**.	-
**1980-1984**	**1/2372**	**4.2**	**0.84 **(0.63; 1.12)	**-16.4% **(-37.4%; 11.6%)	**0.2243**
**1985-1989**	**1/2188**	**4.6**	**0.91 **(0.68; 1.21)	**-9.4% **(-32.0%; 20.7%)	**0.5007**
**1990-1994**	**1/3034**	**3.3**	**0.65 **(0.47; 0.90)	**-34.7% **(-52.6%; -10.0%)	**0.0093**
**1995-1999**	**1/3115**	**3.2**	**0.64 **(0.46; 0.88)	**-36.3% **(-53.8%; -12.3%)	**0.0058**
**2000-2004**	**1/3208**	**3.1**	**0.62 **(0.45; 0.85)	**-38.2% **(-55.0%; -15.1%)	**0.0029**
**2005-2009**	**1/3268**	**3.1**	**0.61 **(0.44; 0.83)	**-39.3% **(-55.8%; -16.7%)	**0.0020**

**Figure 2:**
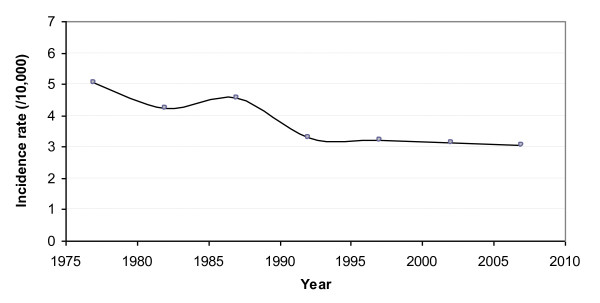
**Evolution of the birth incidence rate of cystic fibrosis in Brittany by 5-year period, over the period 1975-2009**.

Detailed examination of the data revealed a clear breakpoint in the incidence rate at the end of the 1980's. As illustrated in Table 2, the incidence rates of the last four 5-year periods appeared quite similar, and were significantly lower than those of the first three periods. These rates were respectively 1.53, 1.57, 1.62 and 1.65 times lower than that of the oldest period (1975-79). This can also be observed in Figure 2, which shows the evolution of the birth incidence rate by 5-year period.

The most significant change was observed when the year 1990 was considered as the cut-off time. The incidence rate decreased from 4.6/10,000 (i.e. 1/2171) before this year to 3.2/10,000 (i.e. 1/3158) afterwards (corresponding to a decline of 31.3%, p < 0.0001). One can also note that the proportion of sibships with several CF children significantly dropped at the same time (from 9.3% over the period 1975-1989 to 3.2% over the period 1990-2009, p = 0.0094).

The incidence rate has remained quite constant since the early 1990's, since when no significant decline has been observed (annual APC = -1.0%, 95% CI = -3.0% to 1.1%, p = 0.3516).

### Adjusted incidence rate

Finally, we calculated an adjusted incidence rate by taking into account the pregnancy terminations performed after a positive prenatal diagnosis of CF in our area (Table 3).

**Table 3:** Impact of prenatal diagnosis on the incidence rate of cystic fibrosis in Brittany (period 1986-2009).

	Pregnancy terminations	Incidence rate		Variation in incidence
	**No**.	(1/xxxx)	(/10,000)	(%)
**Birth incidence rate of CF**		1/2946	3.4	
**Adjusted birth incidence rate of CF**	105	1/2169	4.6	**35.8%**

Since prenatal diagnosis of CF was done by molecular techniques (i.e. 1986), 105 terminations were performed in such a context among couples living in Brittany, i.e. a mean of four per year. If all these affected pregnancies had resulted in the birth of a CF child, the overall CF incidence rate over the period 1986-2009 would have been 4.6/10,000 (i.e. 1/2169) instead of 3.4/10,000 (i.e. 1/2946), i.e. 35.8% higher.

As shown in Table 4, most of the 105 terminations (60.0%) were done in the parents of previous CF child(ren), 23.8% were done in couples whose 1-in-4 risk was identified following the ultrasound detection of a sign suggestive of CF during pregnancy, and 16.2% were done in 1-in-4 risk couples identified through family testing. Table 4 also shows that, in our area, the impact of the sonographic detection of a sign evocative of CF during pregnancy appeared higher than the impact of family testing.

**Table 4:** Distribution of pregnancy terminations performed in Brittany according to the context of prenatal diagnosis of cystic fibrosis, and assessment of the impact of each strategy on the incidence rate (period 1986-2009).

Context of prenatal diagnosis	Pregnancy terminations		Incidence rate		Variation in incidence
	**No**.	**Freq**.	(1/xxxx)	(/10,000)	(%)
**Parents of a CF child**	63	60.0%	1/2425	4.1	21.5%
**1-in-4 risk couples identified through family testing**	17	16.2%	1/2784	3.6	5.8%
**1-in-4 risk couples identified following ultrasound examination of pregnancy**	25	23.8%	1/2714	3.7	8.5%

**Total**	105	100.0%	1/2169	4.6	**35.8%**

## Discussion

This study deciphers how the incidence of CF has evolved over a long time (the longest ever studied to date: 35 years) in an area where CF is frequent (Brittany, France) and where population-based carrier screening-known to significantly decrease the incidence [12]-is not underway. It also highlights how the incidence is influenced by the health policies implemented.

This study reveals a clear fall in the incidence rate, which dropped by 40% between the period 1975-79 and 2005-09. The observed breakpoint coincides with the more common use of prenatal diagnosis since the end of the 1980's. This test has been increasingly used notably by couples identified to be at risk following NBS, which was implemented in our area in 1989. This study also highlights the importance of ultrasound monitoring performed during pregnancy, which may identify new 1-in-4 risk couples following detection of echogenic bowel.

We have been able to measure accurately the impact of prenatal diagnosis in our area as we have an exhaustive collection of its data. By including pregnancy terminations in the calculations, we have shown that the incidence rate would have been 35.8% higher than that observed to date. The impact of this test appears particularly high in Brittany. Indeed, despite the progress accomplished over the past decades in the field of CF, most 1-in-4 risk couples in our area request prenatal diagnosis and 95% choose not to continue the pregnancy if the fetus is affected [21].

However, the impact of prenatal diagnosis on incidence rate remains relatively limited in areas where population-based carrier screening is not implemented. Indeed, this test can only be offered to 1-in-4 risk couples related to a CF patient or to a carrier child identified through NBS. Therefore, most carrier couples are at risk without knowing it, and can give birth to a CF child. In our area, one individual in 29 is a healthy carrier. Only the implementation of carrier screening may significantly reduce the incidence, as recently shown in an Italian area [12]. Population-based carrier screening consists in offering genetic testing to all the couples planning a pregnancy in a given population. Aware of their high risk, the couples found to have a 1-in-4 risk may opt for prenatal diagnosis and may choose to terminate the pregnancy if the fetus is CF-affected. Such a program is not underway in our area, and this is probably why the incidence has remained stable since the early 1990's.

The present study provides an accurate picture of the evolution of the incidence of CF on the scale of an entire population over a long period. It relies on exhaustive collection of data in a large region whose geographical situation (peninsula) means that the data are well centralized and easier to collect. A few biases may have affected our study. Since 1989, an NBS program has been implemented in our area, which ensures quasi-exhaustive registration of new cases at birth. False-negative cases are always a possibility, but the cases reported so far (which are few in number in the last decade) have been included in the calculations (n = 10). Newborn screening may also diagnose atypical cases that do not meet all the criteria for CF [22,23]. To overcome this problem, which may alter time trends, equivocal diagnoses were excluded from the analysis [15]. Another pitfall that could affect our findings is under-reporting and/or under-diagnosis of cases predating the setting of NBS. The active combination of data sources and the use of capture-recapture studies may have limited this phenomenon. Anyway, in such a situation, the incidence rate would have been still greater in that period, and the observed drop still higher.

Several studies have analyzed time trends in the incidence of CF. While most have reported a decline, some have not observed any temporal change [24]. The findings are however difficult to compare because the studies have been conducted over various periods and in areas that have not implemented similar public health policies and that have different attitudes towards prenatal diagnosis.

The oldest studies were conducted from registry data (prone to under-diagnosis and/or under-reporting), mainly in countries that did not practice NBS. In Canada, time trends were analyzed over the period 1971-2000 and a linear decline was noted since 1988, which coincided with the increased use of family testing and prenatal diagnosis services [6]. In the Netherlands, the incidence rate observed over the period 1974-1997 (2.1/10,000 i.e. 1/4780) was found to be significantly lower than that estimated over a previous period (1961-1965: 2.9/10,000 i.e. 1/3600) [7,25]. In the UK, a constant decline in incidence rate was also observed over a 26-year period (1968-1994) from data of the UK Registry [5].

More recent studies have analyzed time trends in the incidence rate since the implementation of NBS [8]. The region of East Anglia (UK), where NBS has been in place since 1981, experienced a constant decline over the period 1981-1990. In the State of Victoria (Australia), a 17% reduction in the birth incidence of CF was seen following the introduction of NBS in 1989 [26]. The incidence dropped from 3.96/10,000 (i.e. 1/2525) live births over the period 1979-1988 to 3.28/10,000 (i.e. 1/3050) over the period 1989-2006. This was attributed to the uptake of prenatal diagnosis, especially in families identified by NBS. Brittany appears to be similar in various respects, such as the date of implementation of an NBS program, the attitudes towards request of pregnancy terminations, etc. Nevertheless, the decline we observed in our area is rather higher and may, in part, result from the greater uptake of family testing and the efficiency of routine ultrasound monitoring of pregnancy, which is, in our country, supported by our health care system.

In the US, a significant decline has been observed in the States of Wisconsin and of Massachusetts since the implementation of NBS (in 1994 and 1999, respectively) [11,27], whereas no temporal trend has been noted in the State of Colorado, which has been screening newborns for longer (since 1982) [24]. In Massachusetts there has also been reported a change in the composition of the screened cohort (notably a decrease in the proportion of the main F508del/F508del genotype) which coincides with the implementation of carrier screening [11]. As in Colorado, we did not find such a change in our population over time.

A greater decline has recently been found in an area of northern Italy (Veneto/Trentino Alto Adige) over the period 1993-2007, particularly in the region where carrier screening is underway (mean annual decrease of 0.24/10,000) [12]. The authors have also noted a significant correlation between the increase in the number of 1-in-4 risk couples identified and the decline in incidence.

## Conclusion

This study highlights that the time trends observed in the incidence rate depend on the health policies implemented in the studied population (i.e. newborn screening, prenatal diagnosis, carrier screening, etc), but also on the characteristics of the population (i.e. carrier rate, cultural attitudes toward prenatal diagnosis and pregnancy termination, birth rate, etc).

We show, in this study, that the birth incidence of CF has dropped in our area following the implementation of prenatal diagnosis. However, the birth incidence appears now quite stable. The sole strategy that could strongly modify the current incidence of CF would be the routine carrier screening of couples planning a pregnancy. Implementation of such a program has proven successful in reducing the incidence of CF in the State of Massachusetts [11] or in Northern Italy [12], but also the incidence of other diseases, such as thalassemia [28].

Improvement in survival of CF patients as well as in assisted reproduction techniques should help alter the disease dynamics in the future. It is therefore essential to keep on monitoring the incidence to be able to make reliable disease predictions. It is also crucial to maintain and strengthen efforts to improve clinical management and patients' quality of life.

## List of abbreviations

APC: Average percent change; CF: Cystic fibrosis; CFTR: Cystic Fibrosis Transmembrane conductance Regulator; NBS: Newborn screening; OR: Odds-ratio; 95% CI: 95% confidence interval.

## Competing interests

The authors declare that they have no competing interests.

## Authors' contributions

VS and CF conceived and designed the study. VS carried out the statistical analysis and drafted the manuscript. ID managed data collection and participated with PS to data analysis and interpretation. MPA, GR and MR contributed to data collection. All authors revised the manuscript critically for important intellectual content and approved the final version.

## References

[B1] Riordan JR Rommens JM Kerem B Alon N Rozmahel R Grzelczak Z Zielenski J Lok S Plavsic N Chou JL Drumm ML Iannuzzi MC Collins FS Tsui LC Identification of the cystic fibrosis gene: cloning and characterization of complementary DNA Science 1989 245 1066 73 10.1126/science.2475911 2475911

[B2] Welsh MJ Ramsey BW Accurso FJ Cutting GR Scriver CR, Beaudet AL, Sly WS, Valle D, Childs B, Vogelstein B Cystic fibrosis The Metabolic and Molecular Basis of Inherited Disease 2001 8 New York: McGraw Hill 5121 88

[B3] Southern KW Munck A Pollitt R Travert G Zanolla L Dankert-Roelse J Castellani C A survey of newborn screening for cystic fibrosis in Europe J Cyst Fibros 2007 6 57 65 10.1016/j.jcf.2006.05.008 16870510

[B4] Salvatore D Buzzetti R Baldo E Forneris MP Lucidi V Manunza D Marinelli I Messore B Neri AS Raia V Furnari ML Mastella G An overview of international literature from cystic fibrosis registries. Part 3. Disease incidence, genotype/phenotype correlation, microbiology, pregnancy, clinical complications, lung transplantation, and miscellanea J Cyst Fibros 2011 10 71 85 10.1016/j.jcf.2010.12.005 21257352

[B5] Dodge JA Morison S Lewis PA Coles EC Geddes DM Russell G Littlewood JM Scott MT Incidence, population, and survival of cystic fibrosis in the UK, 1968-95 Arch Dis Child 1997 77 493 6 10.1136/adc.77.6.493 9496181 PMC1717408

[B6] Dupuis A Hamilton D Cole DE Corey M Cystic fibrosis birth rates in Canada: a decreasing trend since the onset of genetic testing J Pediatr 2005 147 312 5 10.1016/j.jpeds.2005.06.043 16182667

[B7] Slieker MG Uiterwaal CS Sinaasappel M Heijerman HG van der LJ van der Ent CK Birth prevalence and survival in cystic fibrosis: a national cohort study in the Netherlands Chest 2005 128 2309 15 10.1378/chest.128.4.2309 16236889

[B8] Green MR Weaver LT Heeley AF Nicholson K Kuzemko JA Barton DE McMahon R Payne SJ Austin S Yates JRW Davis JA Cystic fibrosis identified by neonatal screening: incidence, genotype, and early natural history Arch Dis Child 1993 68 464 7 10.1136/adc.68.4.464 8503667 PMC1029265

[B9] Super M Schwarz MJ Malone G Roberts T Haworth A Dermody G Active cascade testing for carriers of cystic fibrosis gene BMJ 1994 308 1462 7 10.1136/bmj.308.6942.1462 8019278 PMC2540318

[B10] Mennie ME Gilfillan A Compton M Curtis L Liston WA Pullen I Whyte DA Brock DJH Prenatal screening for cystic fibrosis Lancet 1992 340 214 6 10.1016/0140-6736(92)90476-J 1353143

[B11] Hale JE Parad RB Comeau AM Newborn screening showing decreasing incidence of cystic fibrosis N Engl J Med 2008 358 973 4 10.1056/NEJMc0707530 18305279

[B12] Castellani C Picci L Tamanini A Girardi P Rizzotti P Assael BM Association between carrier screening and incidence of cystic fibrosis JAMA 2009 302 2573 9 10.1001/jama.2009.1758 20009057

[B13] Scotet V Gillet D Duguépéroux I Audrézet MP Bellis G Garnier B Roussey M Rault G Parent P De Braekeleer M Férec C Réseau mucoviscidose Bretagne et Pays-de-Loire Spatial and temporal distribution of cystic fibrosis and of its mutations in Brittany, France: a retrospective study from 1960 Hum Genet 2002 111 247 54 10.1007/s00439-002-0788-1 12215837

[B14] Scotet V De Braekeleer M Roussey M Rault G Parent P Dagorne M Journel H Lemoigne A Codet JP Catheline M David V Chaventré A Duguépéroux I Verlingue C Quéré I Mercier B Audrézet MP Férec C Neonatal screening for cystic fibrosis in Brittany, France: assessment of 10 years' experience and impact on prenatal diagnosis Lancet 2000 356 789 94 10.1016/S0140-6736(00)02652-0 11022925

[B15] Mayell SJ Munck A Craig JV Sermet I Brownlee KG Schwarz MJ Castellani C Southern KW A European consensus for the evaluation and management of infants with an equivocal diagnosis following newborn screening for cystic fibrosis J Cyst Fibros 2009 8 71 8 10.1016/j.jcf.2008.09.005 18957277

[B16] Dequeker E Stuhrmann M Morris MA Casals T Castellani C Claustres M Cuppens H Des GM Férec C Macek M Pignatti PF Scheffer H Schwartz M Witt M Schwarz M Girodon E Best practice guidelines for molecular genetic diagnosis of cystic fibrosis and CFTR-related disorders-Updated European recommendations Eur J Hum Genet 2009 17 51 65 10.1038/ejhg.2008.136 18685558 PMC2985951

[B17] Castellani C Cuppens H Macek M Jr Cassiman JJ Kerem E Durie P Tullis E Assael BM Bombieri C Brown A Casals T Claustres M Cutting GR Dequeker E Dodge J Doull I Farrell P Férec C Girodon E Johannesson M Kerem B Knowles M Munck A Pignatti PF Radojkovic D Rizzotti P Schwarz M Stuhrmann M Tzetis M Zielenski J Elborn JS Consensus on the use and interpretation of cystic fibrosis mutation analysis in clinical practice J Cyst Fibros 2008 7 179 96 10.1016/j.jcf.2008.03.009 18456578 PMC2810954

[B18] Dobson AJ Barnett AG An introduction to generalized linear models 2008 3 Chapman and Hall/CRC

[B19] Brock DJ Amniotic fluid alkaline phosphatase isoenzymes in early prenatal diagnosis of cystic fibrosis Lancet 1983 2 941 3 6138505 10.1016/s0140-6736(83)90454-3

[B20] McIntosh I Raeburn JA Curtis A Brock DJ First-trimester prenatal diagnosis of cystic fibrosis by direct gene probing Lancet 1989 2 972 3 2571876 10.1016/s0140-6736(89)90974-4

[B21] Scotet V Duguépéroux I Audrézet MP Blayau M Boisseau P Journel H Parent P Férec C Prenatal diagnosis of cystic fibrosis: the 18-year experience of Brittany (western France) Prenat Diagn 2008 28 197 202 10.1002/pd.1910 18240337

[B22] Rosenstein BJ Cutting GR The diagnosis of cystic fibrosis: a consensus statement. Cystic Fibrosis Foundation Consensus Panel J Pediatr 1998 132 589 95 10.1016/S0022-3476(98)70344-0 9580754

[B23] Farrell PM Rosenstein BJ White TB Accurso FJ Castellani C Cutting GR Durie PR Legrys VA Massie J Parad RB Rock MJ Campbell PW III Guidelines for diagnosis of cystic fibrosis in newborns through older adults: Cystic Fibrosis Foundation consensus report J Pediatr 2008 153 S4 S14 10.1016/j.jpeds.2008.05.005 18639722 PMC2810958

[B24] Sontag MK Wagener JS Accurso FJ Sagel SD Consistent incidence of cystic fibrosis in a long-term newborn screen population [abstract] Pediat Pulmonol 2008 43 272 (A203)

[B25] Ten Kate LP Cystic fibrosis in the Netherlands Int J Epidemiol 1977 6 23 34 10.1093/ije/6.1.23 892965

[B26] Massie J Curnow L Gaffney L Carlin J Francis I Declining prevalence of cystic fibrosis since the introduction of newborn screening Arch Dis Child 2010 95 531 3 10.1136/adc.2009.172916 20551198

[B27] Stachiw-Hietpas D Hoffman G Nugent M Schneck K Kennedy-Parker K Roch MJ Farrell PM Simpson P Levy H Wisconsin newborn screening program results suggest a decreasing incidence of CF in the white non-hispanic population [abstract] Pediat Pulmonol 2010 45 390 (A474)

[B28] Cao A Rosatelli MC Monni G Galanello R Screening for thalassemia: a model of success Obstet Gynecol Clin North Am 2002 29 305 28 10.1016/S0889-8545(01)00006-7 12108831

